# The Accuracy of Near Infrared Autofluorescence in Identifying Parathyroid Gland During Thyroid and Parathyroid Surgery: A Meta-Analysis

**DOI:** 10.3389/fendo.2021.701253

**Published:** 2021-06-21

**Authors:** Bin Wang, Chun-Rong Zhu, Hong Liu, Xin-Min Yao, Jian Wu

**Affiliations:** ^1^ Department of Thyroid and Breast Surgery, Chengdu Third People’s Hospital, Chengdu, China; ^2^ Department of Chemistry, School of Basic Medical Science, North Sichuan Medical College, Nanchong, China

**Keywords:** near infrared, autofluorescence, parathyroid gland, surgery, meta-analysis

## Abstract

**Objective:**

We aim to assess the accuracy of near infrared autofluorescence in identifying parathyroid gland during thyroid and parathyroid surgery.

**Method:**

A systematic literature search was conducted by using PubMed, Embase, and the Cochrane Library electronic databases for studies that were published up to February 2021. The reference lists of the retrieved articles were also reviewed. Two authors independently assessed the methodological quality and extracted the data. A random-effects model was used to calculate the combined variable. Publication bias in these studies was evaluated with the Deeks’ funnel plots.

**Result:**

A total of 24 studies involving 2,062 patients and 6,680 specimens were included for the meta-analysis. The overall combined sensitivity and specificity, and the area under curve of near infrared autofluorescence were 0.96, 0.96, and 0.99, respectively. Significant heterogeneities were presented (Sen: I^2^ = 87.97%, Spe: I^2^ = 65.38%). In the subgroup of thyroid surgery, the combined sensitivity and specificity, and the area under curve of near infrared autofluorescence was 0.98, 0.99, and 0.99, respectively, and the heterogeneities were moderate (Sen: I^2^ = 59.71%, Spe: I^2^ = 67.65%).

**Conclusion:**

Near infrared autofluorescence is an excellent indicator for identifying parathyroid gland during thyroid and parathyroid surgery.

## Introduction

During thyroid and parathyroid surgery, identification of the parathyroid gland (PG) always relies on the visual judgment and experience of the surgeon (1, 2). Failure to identify the normal PG during thyroid surgery might result in PG damage, devascularization, and inadvertent resection, and thus bring about postoperative hypoparathyroidism. Hypoparathyroidism can lead to poor experience, prolonged hospitalization, and lower quality of life (3–5). On the other hand, failure to identify the abnormal PG during parathyroid surgery for hyperparathyroidism would result in reoperation (6, 7).

In recent decades, several tracers have been reported to assist to identify PG, but they have some disadvantages, such as lack of direct evidence, limitation of instrument and/or invasion (8–10). Indocyanine green angiography was also used to evaluated the function of PG and showed a satisfactory ability to reduce the rate of hypoparathyroidism after thyroid surgery (11–13). The near infrared autofluorescence (NIRAF), a noninvasive, label-free and rapid indicator, was introduced to intraoperatively identify PG during past years (14–17). The NIRAF of PG was first reported by Paras and his colleagues in 2011 and they found that the fluorescence intensity of PG was greater than that of the thyroid and all other tissues in the neck (18). Since then, the number of studies exploring the potential of NIRAF to identify PG during surgery has being increased and the instruments to measure NIRAF were also various (19–21). Some studies suggested that the use of NIRAF improved the early postoperative hypocalcemia rate and increase parathyroid preservation after total thyroidectomy (22, 23). However, the sensitivity of NIRAF was 81%-100% and the specificity ranged from 80% to 100% (14–16). Due to the large variety, we conduct a meta-analysis to assess the accuracy of NIRAF in identifying PG during thyroid and parathyroid surgery.

## Methods

### Literature Search

Two investigators independently conducted a search by using PubMed, Embase, and the Cochrane Library electronic databases for studies that were published up to 28 February, 2021. The search algorithm was [(Near-infrared) AND (parathyroid)] for PubMed. The following search terms were used in all fields as a search strategy for Embase: 1) parathyroid glands, parathyroid gland, parathyroid, parathyroids; 2) (spectroscopy, near-infrared), near-infrared spectroscopy, near infrared spectroscopy; near infrared, near-infrared. For Cochrane Library electronic databases, the search strategy was the following terms by searching Medical Subject Headings and free word in all field: 1) parathyroid glands, parathyroid gland, parathyroid, parathyroids; 2) (spectroscopy, near-infrared), near infrared spectroscopy, near infrared spectroscopies, near infra-red spectroscopy, near infra-red spectroscopies, near infrared, near infra-red. No restriction was imposed. In addition, we reviewed the reference lists of the retrieved papers and recent reviews.

### Study Selection

The first screening was performed based on the title and abstract, and the full-text was then reviewed. A study was included when it met all the following criteria: 1) NIRAF was used to identify PG; 2) the data to calculate the sensitivity and/or specificity were reported; and 3) aforementioned data showed the numbers of PG. Studies were excluded based on the following criteria: 1) Conference Abstract, Review, Case report, Commentary, Discussion and Letter; 2) those in which the fluorescence originated from tracer; 3) those in which the trial was not conducted in human; 4) those which were published in non-English; 5) those from which data could not be collected adequately; and 6) those that the full text of the studies could not be accessed online or by request to the authors.

### Data Extraction and Quality Assessment

Data were extracted by two reviewers (Wang B and Zhu CR) using a predefined data extraction form, and any disagreement between reviewers was resolved by consensus. Data were collected as follows: the first author, publication time, type of study, country of origin, study sites and institutes, measurement instrument and method, research period, sample size, the age of patients, disease (the reason for surgery) and surgical method, diagnostic standard and reference standard of PG, data to calculate the sensitivity and/or specificity. The quality of study was assessed with the Quality Assessment of Diagnostic Accuracy Studies (QUADAS-2) (24).

### Statistical Analysis

Combined sensitivity, combined specificity and summary receiver operating characteristic (SROC) curve were used to investigate the accuracy with Stata version 14.0 (Stata Corp LP, College Station, Texas, USA). The quality assessment of study was achieved through Review Manager 5.4. Meta-regression analysis was performed with removing the covariate with the largest P-value one by one by Meta-Disc 1.4. Heterogeneity was quantified statistically with the I^2^ test. P < 0.1 and I^2^ > 50% for heterogeneity were considered significant differences. We explored the reasons for heterogeneity by performing subgroup analyses. The measurement instrument, disease type and reference standard were examined as the potential influence factors on the accuracy of NIRAF. Potential publication bias was assessed by the Deeks’ funnel plots. P < 0.05 was considered statistically significant.

## Results

### Literature Search

**Figure 1:**
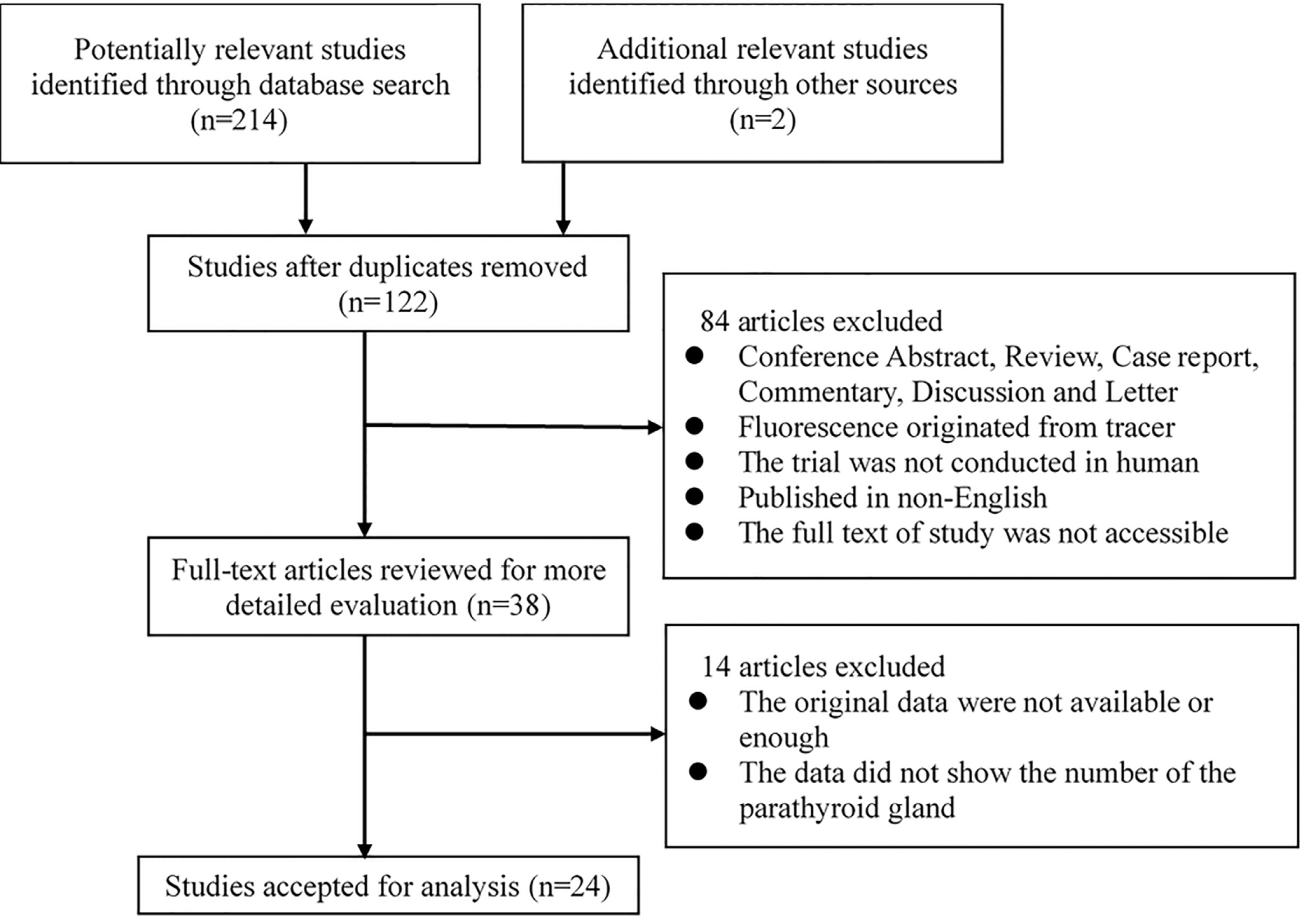
Flow chart of study selection.

The study selection process is shown in **Figure 1**. A total of 214 potentially relevant records were identified through searching these databases and 2 other records were added by reviewing the reference lists of the retrieved papers. And one hundred and twenty-two records were retained after duplicates were removed. After screening the titles and abstracts, 84 studies were excluded for various reasons. The remaining 38 studies were assessed *via* full-text screening, and 14 studies were further excluded. Finally, 24 independent studies were included in the meta-analysis (14–16, 19–21, 25–42).

### Study Characteristics

**Figure 2:**
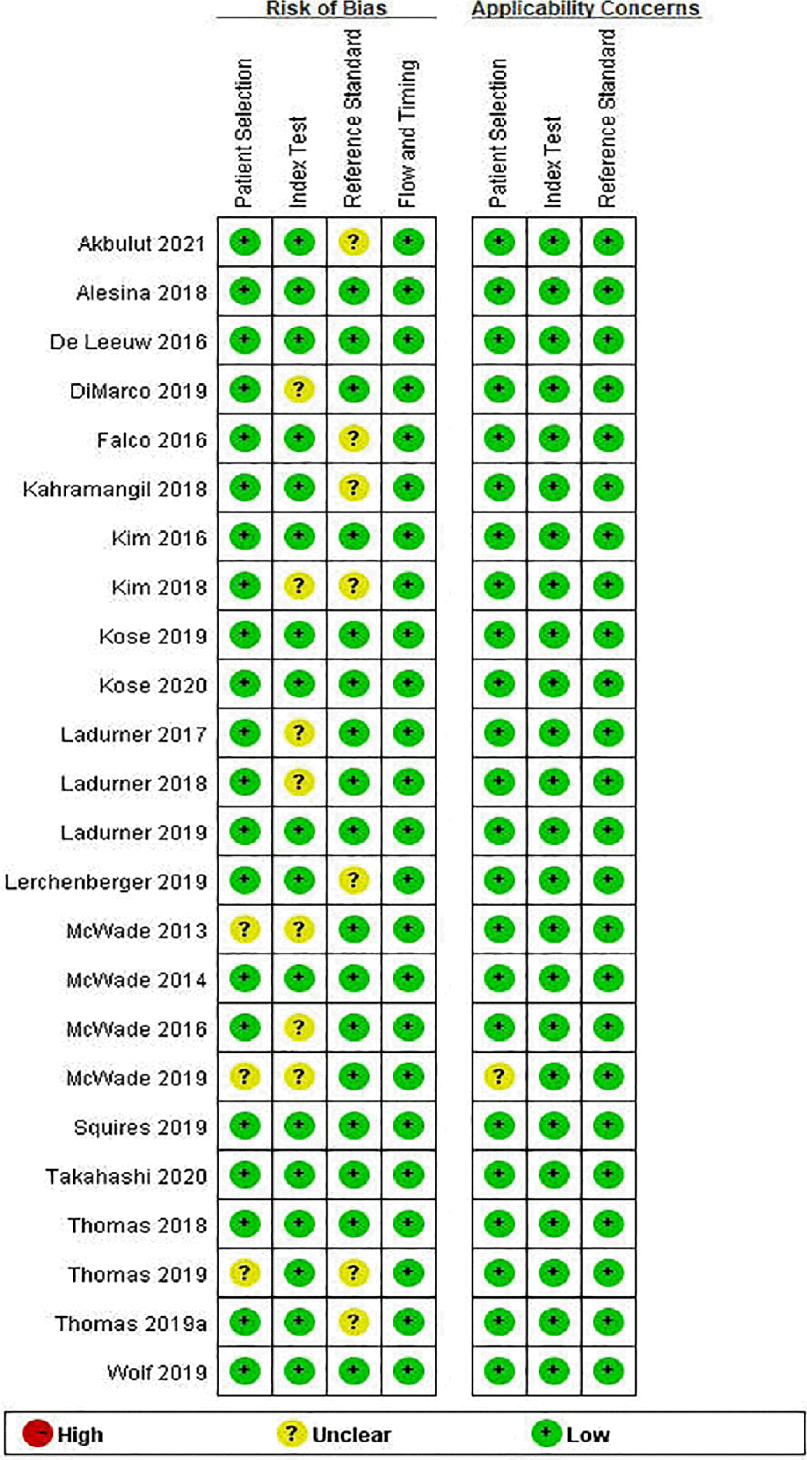
Risk of bias and applicability concerns summary: review authors’ judgements about each domain for each included study.

**Table 1** summarizes the basic information of the 24 included eligible studies (14–16, 19–21, 25–42). These studies were published between 2013 and 2020 and all of them were prospective studies except one (16). Of the 24 studies, 3 were conducted in Asia, 8 in Europe, 11 in the United States, 1 in Argentina, and 1 in multicenter of American, France and Argentina. According to these studies, 11 types of instrument based on NIRAF were used to identify PG and a total of 2,062 patients and 6,680 specimens were included for analysis. The quality assessment of these studies was shown in **Figure 2**.

**Table 1:** The characteristics of the included studies.

Study ID	First author	Publication time	Type of study	Country/Region	Institute	Instrument	Measurement method	Research period	Patients(n)	Age (Mean ± Standard, Range)	Diagnostic standard	Disease	Surgerial method	Reference standard
**1**	McWade	2013	Prospective	American	Vanderbilt University	Fluorescence spectroscopy system (spectrometer; S2000-FL; Ocean Optics, Dundelin, FL)	contact	NA	45	NA	P/T:1.2	T, pHPT, sHPT	Tx, PTx	Experience, Pathology reports
**2**	McWade	2014	Prospective	American	Vanderbilt University	Fluorescence spectroscopy system (spectrometer; S2000-FL; Ocean Optics, Dundelin, FL)	contact	NA	110	NA	P/T:1.2	T, pHPT, sHPT	Tx, PTx	Experience, Pathology reports
						Modified NIR imaging system (Karl Storz, PDD Camera El Segundo, CA)	15cm	NA	3	NA	Y	T, pHPT, sHPT	Tx, PTx	Experience, Pathology reports
**3**	De Leeuw	2016	Prospective	France	Universite´ Paris-Saclay	Fluobeam (Fluoptics, Grenoble, France)	20cm	2014.12-2015.3	63	NA	Y	T, HPT	Tx, PTx	Experience, Pathology reports
**4**	Falco	2016	Prospective	Argentina	University of Buenos Aires;Cleveland Clinic Florida	Fluobeam (Fluoptics, Grenoble, France)	20cm	2015.6-2015.8	28	NA	P/B:1	T, pHPT	TTx, PTx	Experience, Pathology reports
**5**	Kim	2016	Prospective	South Korea	Kosin University	Fluorescence imaging devices (EOS REBEL T3, Canon, Tokyo, Japan)	NA	NA	8	34-73	P/B:1	T	Tx	Experience
**6**	McWade	2016	Prospective	American	Vanderbilt University	Fluorescence spectroscopy system (spectrometer; S2000-FL; Ocean Optics, Dundelin, FL)	contact	NA	137	53, 20–87	P/T:1.2	T, HPT	Tx, PTx	Experience, Pathology reports
**7**	Ladurner	2017	Prospective	Germany	Ludwig Maximilians University Munich	Near-infrared system (Tricam SL II, Karl Storz GmbH &Co KG, Tuttlingen, Germany)	5cm	NA	30	NA	Y	T, HPT	Tx, PTx	Experience, Pathology reports
**8**	Alesina	2018	Prospective	Germany	Universität Duisburg-Essen	Modular IMAGE1 S™ Camera (Karl Storz Endoskope, Tuttlingen, Germany^®^)	NA	2017.7-2017.8	5	56.2 ± 12.4, 45-77	Y	T, pHPT	TT, PTx	Experience, Pathology reports
**9**	Kahramangil	2018	Prospective	American, France, Argentina	Cleveland Clinic Foundation, OH;Hoˆpital Europe´en, Marseille, France;Buenos Aires	Fluobeam (Fluoptics, Grenoble, France)	20cm	NA	210	53.1 ± 14	P/B:1	T, pHPT	Tx, PTx	Experience, Pathology reports
**10**	Kim	2018	Prospective	South Korea	Kosin University	Fluorescence imaging devices (EOS REBEL T3, Canon, Tokyo, Japan)	NA	2015.7-2017.1	38	19-73	Y	T	TTx, LTx	Experience
**11**	Ladurner	2018	Prospective	Germany	Ludwig Maximilians University Munich	Near-infrared system (Tricam SL II, Karl Storz GmbH &Co KG, Tuttlingen, Germany)	5cm	NA	20	NA	Y	T	Tx	Experience, Pathology reports
**12**	Thomas	2018	Prospective	American	Vanderbilt University	Fluorescence spectroscopy system (spectrometer; S2000-FL; Ocean Optics, Dundelin, FL)	contact	NA	162	NA	P/T:1.2	T, HPT	TT, PTx	Experience, Pathology reports
						PTeye	contact	NA	35	NA	P/T:1.2	T, HPT	TT, PTx	Experience, Pathology reports
**13**	DiMarco	2019	Prospective	UK	Hammersmith Hospital	Fluobeam (Fluoptics, Grenoble, France)	20cm	2017.2-2017.10	96	24-85	P/T:1	HPT	PTx	Experience, Pathology reports
**14**	Kose	2019	Prospective	American	Cleveland Clinic, Cleveland, OH	Fluobeam (Fluoptics, Grenoble, France)	20cm	2016.7-2018.2	50	59.6 ± 12.4	Y	pHPT	PTx	Experience, Pathology reports
**15**	Ladurner	2019	Prospective	Germany	Ludwig Maximilians University Munich	Near-infrared system (Tricam SL II, Karl Storz GmbH &Co KG, Tuttlingen, Germany)	5cm	2014.10-2019.4	117	49.9, 19-81	Y	T, pHPT	Tx, PTx	Experience, Pathology reports
**16**	Lerchenberger	2019	Prospective	Germany	Ludwig Maximilians University Munich	Near-infrared system (Tricam SL II, Karl Storz GmbH &Co KG, Tuttlingen, Germany)	5cm	2017.10-2018.5	50	47.2	Y	T, HPT	Tx, PTx	Experience, Pathology reports
**17**	McWade	2019	Prospective	American	Vanderbilt University	OTIS/Basler AG, Ahrensburg, Germany	35cm	NA	30	56.1 ± 11.7, 29-74	P/B:1.5	T, pHPT, sHPT	Tx, PTx	Experience, Pathology reports
**18**	Squires	2019	Prospective	American	The Ohio State University Wexner Medical Center	PDE-Neo II (Hamamatsu, Mitaka USA, Inc)	5cm	2017.6-2018.2	59	NA	P/T:1.1	pHPT	PTx	Pathology reports
**19**	Thomas	2019	Prospective	American	Vanderbilt University	PTeye	contact	NA	20	46.8 ± 14.2, 21-70	P/T:1.2	T, pHPT, sHPT	TT, PTx	Experience, Pathology reports
						Modified NIR imaging system (Karl Storz, PDD Camera El Segundo, CA)	15cm	NA	6	54.8 ± 17.6, 29-75	Y	T, pHPT	TT, PTx	Experience, Pathology reports
						OTIS/Basler AG, Ahrensburg, Germany	50cm	NA	15	55.1 ± 13.1, 29-70	P/B:1.5	T, pHPT, sHPT	TT, PTx	Experience, Pathology reports
**20**	Thomas	2019	Prospective	American	Vanderbilt University	PDE-Neo II (Hamamatsu, Mitaka USA, Inc)	5cm	2018.12-2019.1	20	NA	P/B:1.1	T, pHPT	TT, PTx	Experience, Pathology reports
						PTeye	contact	2018.12-2019.1	20	NA	P/T:1.2	T, pHPT	TT, PTx	Experience, Pathology reports
**21**	Wolf	2019	Retrospective	Germany	Schwarzwald-Baar Klinikum Villingen-Schwenningen	Near-infrared system (Tricam SL II, Karl Storz GmbH &Co KG, Tuttlingen, Germany)	2-3cm	2012.2-2019.9	39	NA	Y	HPT	PTx	Pathology reports
**22**	Akbulut	2020	Prospective	American	Cleveland Clinic, Cleveland, Ohio	Fluobeam (Fluoptics, Grenoble, France)	20cm	2016.7-2020	200	54.5	P/B:1	T, HPT	Tx, PTx	Experience, Pathology reports
						Fluobeam LX (Fluoptics, Grenoble, France)	20cm	2016.7-2020	100	53.4	P/B:1	T, HPT	Tx, PTx	Experience, Pathology reports
**23**	Kose	2020	Prospective	American	Cleveland Clinic, OH	Fluobeam (Fluoptics, Grenoble, France)	20cm	2016.7-2018.10	310	55.6 ± 15.2	P/B:1	T, pHPT	Tx, pPTx	Experience, Pathology reports
**24**	Takahashi	2020	Prospective	Japan	Niigata University	PDE-Neo,Hamamatsu Photonics, Hamamatsu, Japan	5cm	2019.7-2019.12	36	61 ± 15.4	P/T:1	T	Tx	Pathology reports

NA, Not Available; P/T, the ratio of fluorescence intensity for parathyroid/thyroid; P/B, the ratio of fluorescence intensity for parathyroid/background; Y, fluorescence was obtained; T, thyroid disease; HPT, hyperparathyroidism; pHPT, primary hyperparathyroidism; sHPT, secondary hyperparathyroidism; Tx, thyroidectomy (hemi- or total); TTx, total thyroidectomy; PTx, parathyroidectomy.

### The Accuracy of NIRAF in Identifying PG

**Figure 3:**
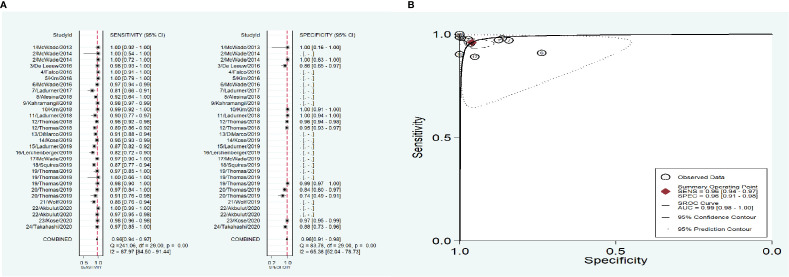
The accuracy of near infrared autofluorescence. **(A)** forest plot of the included studies. **(B)** summary receiver operating characteristic (SROC) curve.

As shown in **Figure 3**, the overall combined sensitivity and specificity were both 0.96, and the area under curve (AUC) was 0.99. Significant heterogeneities were detected (Sen: I^2^ = 87.97%, Spe: I^2^ = 65.38%), but the publication bias did not appear significant as measured by the Deeks’ funnel plot asymmetry tests (p=0.86).

**Figure 4:**
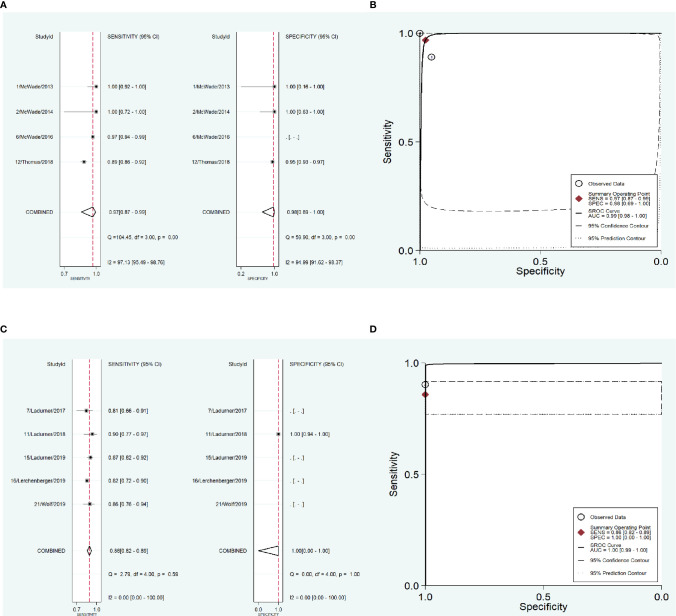
The accuracy of near infrared autofluorescence in the subgroup analysis of the measurement instrument. **(A)** forest plot of the studies in the subgroup of fluorescence spectroscopy system (spectrometer; S2000-FL; Ocean Optics, Dundelin, FL). **(B)** summary receiver operating characteristic (SROC) curve in the subgroup of fluorescence spectroscopy system (spectrometer; S2000-FL; Ocean Optics, Dundelin, FL). **(C)** forest plot of the studies in the subgroup of near infrared system (Tricam SL II, Karl Storz GmbH &Co KG, Tuttlingen, Germany). **(D)** summary receiver operating characteristic (SROC) curve in the subgroup of near infrared system (Tricam SL II, Karl Storz GmbH &Co KG, Tuttlingen, Germany).

**Figure 5:**
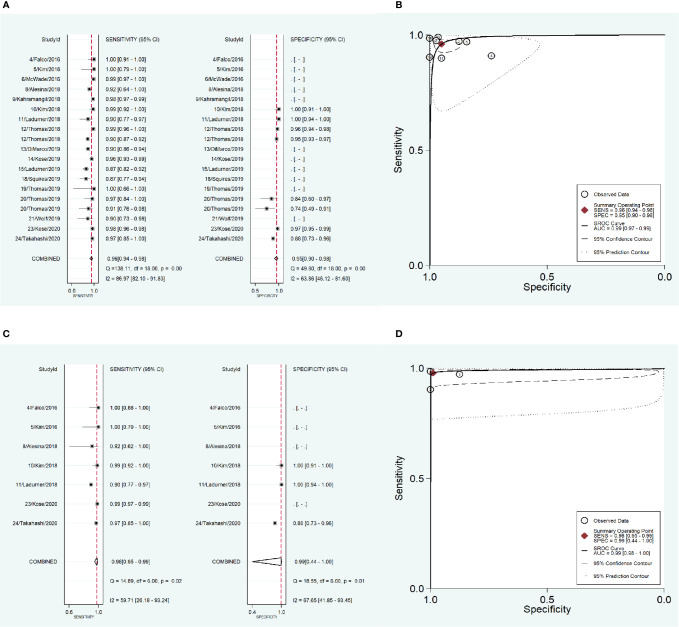
The accuracy of near infrared autofluorescence in the subgroup analysis of the type of disease. **(A)** forest plot of the studies in the subgroup of primary hyperparathyroidism and thyroid disease. **(B)** summary receiver operating characteristic (SROC) curve in the subgroup of primary hyperparathyroidism and thyroid disease. **(C)** forest plot of the studies in the subgroup of thyroid disease. **(D)** summary receiver operating characteristic (SROC) curve in the subgroup of thyroid disease.

**Figure 6:**
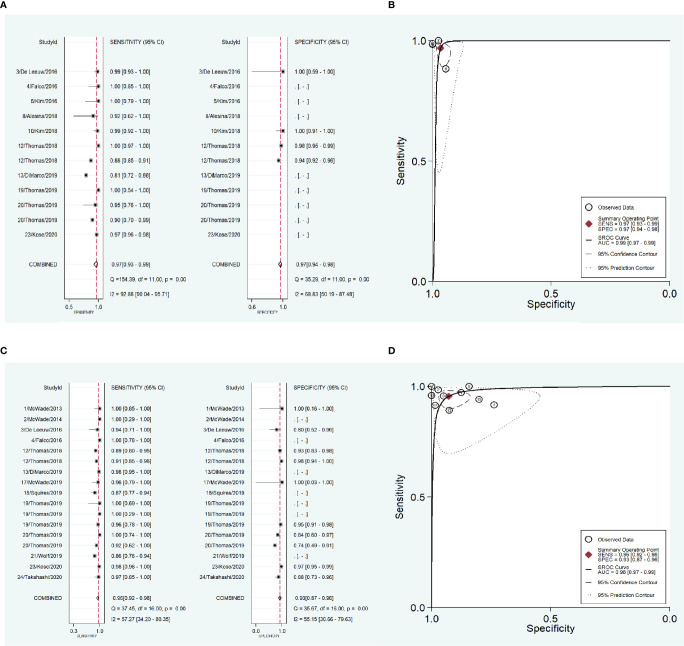
The accuracy of near infrared autofluorescence in the subgroup analysis of the reference standard of the diagnosis of parathyroid gland. **(A)** forest plot of the studies in the subgroup of experience. **(B)** summary receiver operating characteristic (SROC) curve in the subgroup of experience. **(C)** forest plot of the studies in the subgroup of pathology report. **(D)** summary receiver operating characteristic (SROC) curve in the subgroup of pathology report.

To address the heterogeneity, subgroup analysis was performed according to the measurement instrument, disease and reference standard. When the fluorescence spectroscopy system (spectrometer; S2000-FL; Ocean Optics, Dundelin, FL) was used, the combined sensitivity and specificity increased (Sen: 0.97, Spe: 0.98; **Figure 4A**) and the AUC was also 0.99 (**Figure 4B**), but the heterogeneities were still significant (Sen: I^2^ = 97.13%, Spe: I^2^ = 94.99%; **Figure 4A**). When the near infrared system (Tricam SL II, Karl Storz GmbH &Co KG, Tuttlingen, Germany) was used, although the heterogeneities decreased, the combined sensitivity was also decreased, and the combined specificity and AUC were not able to be combined because of lack of data (**Figures 4C, D**). When NIRAF was used during thyroid surgery and parathyroid surgery for primary hyperparathyroidism, the combined specificity decreased, but the combined sensitivity and AUC were same as the overall results and the heterogeneities did not also obviously change (Sen: 0.96, I^2^ = 86.97%, Spe: 0.95, I^2^ = 63.86%, AUC=0.99; **Figures 5A, B**). However, when NIRAF was used to identify normal PG during thyroid surgery, the combined sensitivity and specificity both increased (Sen: 0.98, Spe: 0.99; **Figure 5C**) and the AUC kept stable (AUC=0.99, **Figure 5D**), but the heterogeneities decreased (Sen: I^2^ = 59.71%, Spe: I^2^ = 67.65%; **Figure 5C**). When subgroup analysis was performed according to the reference standard, the combined sensitivity, combined specificity and AUC were respectively 0.97, 0.97 and 0.99 in the subgroup of experience (**Figures 6A, B**), and 0.96, 0.93 and 0.98 in the subgroup of pathology report (**Figures 6C, D**). The heterogeneities increased in the subgroup of experience (Sen: I^2 =^ 92.88%, Spe: I^2^ = 68.83%, **Figure 6A**), while they reduced in the subgroup of pathology report (Sen: I^2^ = 57.27%, Spe: I^2^ = 55.15%, **Figure 6C**).

Although the heterogeneities got diminished in the subgroup of near infrared system (Tricam SL II, Karl Storz GmbH &Co KG, Tuttlingen, Germany), thyroid surgery and pathology report, they were still significant. We further performed meta-regression and found that the type of disease was related to heterogeneity (P = 0.01, **Table 2**).

**Table 2:** The result of meta-regression.

Variable	Coefficient	P	RDOR (95%CI)
**Covariate**	1.553	0.3054	
**S**	-0.187	0.4366	
**Disease**	1.126	0.0123	3.08 (1.32;7.18)

## Discussion

The present meta-analysis demonstrated that NIRAF had a high accuracy in identifying PG during thyroid and parathyroid surgery, and the measurement instrument, status of PG and the reference standard of diagnosis of PG would influence the identification accuracy.

Some studies suggested that the accuracy of NIRAF in identify PG was closed to 100% (30, 41, 42). While, some other researchers got the result that the accuracy was less than 85% (15, 16). The difference in measurement instruments might be a reason for the difference in accuracy and the high heterogeneity across these studies. As described by Solorzano (43), all the approaches for NIRAF detection were based on probe or image, and different approaches had respective advantages and disadvantages, and thus these characteristics of different approaches might result in the difference in the identification accuracy. Due to the lack of data, the subgroup analysis of measurement instrument was only performed in two types of instrument. And we found that the heterogeneity decreased in the subgroup of near infrared system (Tricam SL II, Karl Storz GmbH &Co KG, Tuttlingen, Germany). Di Marco (44) and Solorzano (45) also reviewed the studies that NIRAF was used in thyroid and parathyroid surgery, but they did not perform the meta-analysis.

On account of the subjectivity of experience, the reference standard of diagnosis of PG was also regarded as an influence factor to be performed subgroup analysis. In the subgroup where pathology report was considered as reference standard, the heterogeneity diminished significantly. Although the surgeons in these studies were all senior professional endocrine surgeons, their experience and ability to identify PG might not be still fully consistent.

Surgical method depends on the type of disease, which means that PGs was normal in thyroid surgery and was hyperplastic in parathyroid surgery for primary or/and secondary hyperparathyroidism. When we performed subgroup analysis according to the type of disease, the heterogeneity became moderate in the subgroup of thyroid disease. And the meta-regression analysis also confirmed that the type of disease was significantly related to the heterogeneity. McWade et al. (25) and Kose et al. (34) found that the fluorescence intensity of hyperplastic PG was larger than that of normal PG, while DiMarco (33) and Squires (38) suggested that there was no significant difference in fluorescence intensity between hyperplastic PG and normal PG, but Falco (26) and Aoyama (46) reported that the fluorescence of hyperplastic PG was weaker than that of normal PG. The inconsistent results of comparison of fluorescence intensity between hyperplastic PG and normal PG might affect PG identification and thus cause the heterogeneity. The reason for the difference in fluorescence intensity of hyperplastic PG might be that the fluorescence intensity of PG with primary hyperparathyroidism was stronger than that of PG with secondary hyperparathyroidism and the fluorescence intensity distributed unevenly in hyperplastic PG (47). Wolf and colleagues reported a lower accuracy in identifying PG during surgery for secondary hyperparathyroidism than that during surgery for primary hyperparathyroidism (16). However, we could not verify this result, because the subgroup analyses were not able to be performed in the subgroup of primary and secondary hyperparathyroidism on account of lack of data.

Several limitations existed in this meta-analysis. First, data of sensitivity and specificity could not be collected at the same time in some studies. Second, although we performed subgroup analysis, the heterogeneity did not disappear. Third, some subgroup analyses could not be performed because of lack of eligible data. Fourth, the diagnostic standards were not uniform among these studies, even though no threshold effect was observed in this meta-analysis. Lastly, the exclusion of non-English-language studies might lead to bias.

## Conclusion

In conclusion, the NIRAF is an excellent indicator for identifying PG during thyroid and parathyroid surgery, and the accuracy is perfect, especially in thyroid surgery. The ability of NIRAF to preserve PG function during thyroid surgery is worth exploring.

## Data Availability Statement

The original contributions presented in the study are included in the article/supplementary material. Further inquiries can be directed to the corresponding author.

## Author Contributions 

Study conception and design: BW, C-RZ, HL, X-MY, and JW. Acquisition of data: BW and C-RZ. Analysis and interpretation of data: BW, C-RZ, and HL. Drafting of manuscript: BW and C-RZ. Critical revision: BW, C-RZ, HL, X-MY, and JW. All authors contributed to the article and approved the submitted version.

## Funding

BW was supported by a nonprofit fund from CHINA HEALTH PROMOTION FOUNDATION. JW was supported by a grant from Scientific Research Fund of the Department of Science and Technology of Chengdu City (2015-HM01-00376-SF) and Science and Technology Program of Science & Technology Department of Sichuan Province (2015JY0190). The funding bodies had no role in the conception of the study, in the collection, analysis, and interpretation of data, in writing the manuscript and in the approval of the publication.

## Conflict of Interest

The authors declare that the research was conducted in the absence of any commercial or financial relationships that could be construed as a potential conflict of interest.
